# Study protocol of the multi-site randomised controlled REDALI-DEM trial - The effects of structured Relearning methods on Daily Living task performance of persons with Dementia

**DOI:** 10.1186/1471-2318-11-44

**Published:** 2011-08-18

**Authors:** Sebastian Voigt-Radloff, Rainer Leonhart, Marcel Olde Rikkert, Roy Kessels, Michael Hüll

**Affiliations:** 1 University Hospital Freiburg, Centre of Geriatric Medicine and Gerontology Freiburg, Lehener Strasse 88, 79106 Freiburg, Germany; 2 University of Freiburg, Psychological Institute, Department of Social Psychology & Methodology, Engelberger Str. 41, 79085 Freiburg, Germany; 3 Radboud University Nijmegen Medical Centre, Geriatric Department, PO Box 9101, 6500 HB Nijmegen, The Netherlands; 4 Radboud University Nijmegen Medical Centre, Department of Medical Psychology & Donders Institute for Brain, Cognition and Behaviour, PO Box 9101, 6500 HB Nijmegen, The Netherlands

## Abstract

**Background:**

Evidence from pilot trials suggests that structured learning techniques may have positive effects on the performance of cognitive tasks, movement sequences or skills in patients with Alzheimer's disease. The purpose of this trial is to evaluate whether the usual method of learning by trial and error or the method of errorless learning demonstrate better effects on the performance of two selected daily living tasks six weeks after the intervention in people with mild to moderate dementia.

**Methods/Design:**

A seven-centre single-blind, active-controlled design with a 1:1 randomisation for two parallel groups will include 175 persons diagnosed with Alzheimer's disease or mixed type dementia (MMSE 14-24), living at home, showing at least moderate need for assistance in instrumental activities of daily living; primary carer available and informed consent of patient and primary carer. Patients of both study arms will receive 15 one-hour-sessions at home by trained interventionists practising two daily living tasks individually selected. In one group the trial and error technique and in the other group the errorless learning method will be applied. Primary outcome is the task performance measured with the Task Performance Scale six weeks post treatment.

**Discussion:**

The trial results will inform us to improve guidelines for instructing individuals with memory impairments. A user-friendly practice guideline will allow an efficient implementation of structured relearning techniques for a wide range of service providers in dementia care.

**Trial registration:**

DRKS00003117

## Background

Dementia is a neurodegenerative progressive disease with high prevalence and incidence rates and enormous costs for international societies [1-7]. Consequences of dementia are an increasing deterioration of patients' daily functioning and a high burden mainly borne by the primary carers or nursing staff in nursing homes and in the community [8-10]. Relearning of relevant daily-living tasks in dementia has the potential to stabilise the daily functioning of people with dementia and to increase their independency. Alzheimer's Disease (AD) alters memory and learning to such a degree that it heavily interferes with daily living. Normally, learning occurs in an unstructured manner by trial and error, which consists of guessing and the occurrence of errors during acquisition. Errorless learning is a teaching technique using feed-forward instruction, in order to prevent people from making mistakes during the learning process. In AD patients, the amount of such errors may be reduced by providing adapted cues prior to performing the target task and limiting the patient's guessing [11].

Evidence from small-scaled pilot trials suggests that errorless learning techniques may have positive effects on the performance of cognitive tasks, movement sequences or skills in patients with AD [11-17]. A meta-analysis found large effect sizes in favour of errorless learning (d = 0.87, p = 0.008) [18]. The Nijmegen research group compared the effects of learning strategies on activities of daily living in a pilot trial with 12 AD patients from nursing homes and day care centres. Patients in the errorless learning group significantly improved their performances with large effect size after six sessions (η^2^_p _= 0.8, p < 0.01) and at delayed assessment (η^2^_p _= 0.5, p = 0.01) [19]. These preliminary data show that people with AD can relearn tasks they were not able to perform before. However, the results need to be replicated at a larger scale and within the patient's familiar environment.

Therefore, the purpose of our multi-centre REDALI-DEM study is to evaluate whether the usual method of learning by trial and error (TEL) or the method of errorless learning (ELL) demonstrate better effects on the performance of two selected daily living tasks six weeks after the intervention in people with mild to moderate dementia living at home. Secondary research questions are:

• Can effects on performance be maintained 3 months post treatment?

• Does relearning of two relevant daily living tasks show any generalising effect on the patients' initiative or need for assistance in activities of daily living?

• Costs and practicability of as well as patients' satisfaction with treatment.

## Methods/Design

To compare the effects of errorless and trial and error learning, we used a seven-centre single-blind, active-controlled design with a 1:1 randomisation for two parallel groups. The study has been registered at the German Trial Register (No. DRKS00003117, available at http://apps.who.int/trialsearch). The Medical Ethics Committee of the University Hospital Freiburg has given ethical approval (No. 194/11).

### Participants and setting

#### Inclusion criteria

• Persons diagnosed with Alzheimer's disease or mixed type dementia.

• Mini Mental State Examination: 14-24.

• Living at home.

• Carer available for rating the need for assistance in activities of daily living.

• At least moderate need for assistance in instrumental activities of daily living; mean score of the five household items in the performance scale of the Interview for Deterioration in Daily Living Activities in Dementia must be 2 or higher.

• Informed consent of patient and primary carer.

#### Exclusion criteria

• Major depression, Geriatric Depression Scale (15 items) ≥ 9.

• Major need of physical nursing care (level 2 or higher according to the German Long-Term Care Insurance Act indicated by 120 min or more per day).

• Severe behavioural disturbances, unstable medical conditions or lack of attention and understanding of simple task instructions in German language, which do not allow participation in the study as judged by the recruiting study physician.

• Involvement in other clinical trials.

• In order to achieve the recruitment of a naturalistic sample, there will be no limitation on drug therapy. During the trial duration, drug treatment should be kept as constant as it is appropriate for an optimal routine medication management. Within the statistical analysis, we will control for possible changes in anti-dementia drug treatment in both groups.

#### Setting

REDALI-DEM study centres are five outpatient memory centres at university hospitals in Bonn, Freiburg, Mainz, Marburg and Tübingen; one Department of a municipal hospital in Stuttgart specialising in geronto-psychiatry; and a centre for psychiatry in Emmendingen. The centres are located throughout Germany in urban regions and have all provided outpatient dementia care for five to seventeen years. Their standard service comprises diagnostic work-up for dementia and related diagnoses as well as recommendation of risk reduction, dementia medication and non-pharmacological treatments. Principal investigators of the centres are psychiatrists or neurologists with eight to fifteen years of experience in dementia care. We will control for possible site effects.

### Intervention

#### Intervention scheme

1. The scheme for the ELL and TEL intervention is divided into two blocks (table 1). The first block consists of 12 sessions within 10 weeks, the second block includes 3 refresher sessions within two weeks.

**Table 1:** Intervention scheme

Weeks	0	1-10	11	16*	19-20	26
Measurement time points	**t**_**0**_		**t**_**1**_	**t**_**2**_		**t**_**3**_
Intervention		12 sessions		break	3 refresher sessions	

* Primary measurement time point

2. In the first block, the general scheme is 2 sessions per week, with following exceptional cases: When patient is ill or not available, protocol allows

a. maximum 4 weeks without session in total

b. maximum 3 sessions per week are allowed to catch up on the missing sessions

3. In the second block, 3 refresher sessions must be performed within two weeks.

4. In order to avoid contamination, ELL and TEL interventions are delivered by separate interventionists and the seminars on ELL and TEL interventions are separated.

5. Each study-site has three interventionists, in order to avoid that more than 21 interventionists are to be instructed. At least one interventionist must be an occupational therapist. The other interventionists can be trained from another professional background in the medical field, e.g. psychology or nursing. Their curriculum vitae must indicate professional experience with or special qualification for geronto-psychiatric patients. Each site has one main ELL interventionist, one main TEL interventionist and one substitute interventionist, who must substitute both, the ELL and TEL interventionist. The ELL interventionist is not allowed to attend the seminar on the TEL intervention and vice versa. The substitute interventionists must attend both seminars, TEL and ELL. Maximal 5 treatment sessions per patient (33%) can be applied by the substitute, in order to reduce the contamination rate.

6. In order to reduce bias by systematic selection, interventionists of each study site are assigned by random to be ELL or TEL interventionist.

#### Intervention sessions

Baseline session (same procedure in ELL and TEL group)

1. Selection of two tasks

Together with the patient and the carer, the interventionist determines two tasks to be relearned based on following criteria:

a. Tasks are from a pre-structured pool of IADL tasks, such as changing batteries, folding a t-shirt, setting a table, using a coffee machine. This pool consists of 20 IADL tasks, each of it structured in single task steps.

b. The patient and the carer confirm that the patient (1) was used to regularly perform the tasks within the last year, (2) that he is not able to do so anymore and (3) that he is still interested in. It must be clear that the patient himself is motivated to do the tasks again and that this motivation is not primarily driven by the carer's interest.

c. The interventionist asks the patient to try to perform the tasks without cue and can clearly observe that the patient is not able to perform the tasks without errors.

2. Video tapes of baseline task performance

a. Task I: The interventionist asks the patient to perform task I. The interventionist video records the performance without giving any verbal cue or instruction.

b. Task II: The same procedure.

Relearning sessions are scheduled for a total of 120 min. It is not intended to involve the carer during the relearning sessions.

1. The **ELL **condition consists of the following procedures.

a. The interventionist provides the patient with a special cue card series depicting each task step.

b. He asks the patient to perform the task step by step.

c. As soon as the interventionist anticipates that the patient could make an error, he intervenes and again gives brief verbal instructions or short demonstration as cues for the correct performance.

d. When the patient has performed the first step correctly, the interventionist gives instruction for the next step.

e. These procedures of interventionist's instruction, patient's performance and interventionist's early intervening for avoiding errors are followed until the whole task is performed.

f. However, the training will stop after 30 min, no matter how many steps of the task the patient has performed without error. When the patient shows errorless performance of the whole task in less than 30 min training, the same task is trained again, but not more than twice.

2. The **TEL condition **consists of the following procedures.

a. The interventionist asks the patient to perform the task. Cue cards are not provided.

b. When the patient makes an error, he is allowed up to three guesses (or a maximum of 30 seconds without guess) to correct on his own.

c. If the participant is unable to perform the step correctly, the interventionist will use different questions relative to the purpose of the task, in order to help the patient to find solutions.

d. If the participant is still unable to perform the step correctly, the interventionist will give the correct verbal instruction - but no demonstration - of what is to do next, so that the patient can perform the step correctly.

e. These procedures of patient's performance incl. guessing, interventionist's helping questions and - if necessary - correct instructions are followed until the whole task is performed.

f. The training will stop after 30 min, no matter how many steps of the tasks the patient has performed. When the patient performed the task in less than 30 min training, the same task is trained again, but not more than twice.

Final intervention session (same procedure in ELL and TEL group)

Video tapes of baseline task performance

a. Task I: The interventionist again video records the performance without giving any cue or instruction.

b. Task II: The same procedure.

Assessment sessions

The interventionist must video record the patient's task performance at measurement time point T_2 _(16 weeks after baseline) and T_3 _(26 weeks after baseline). In the assessment visits, he has to follow the same procedures as in the final intervention session.

#### Intervention adherence

Interventionists of both treatment conditions will be introduced during a seminar of four days, perform training sessions with two pilot patients from their study site within the next 4 months and subsequently attend a repetition seminar of two days. Methods of the seminar are presentations, role-plays and feedback rounds on role-plays of each interventionist. Experts in relearning in AD from Nijmegen will give two separate seminars for the ELL and TEL arm.

In both seminars interventionists will be instructed in

1. understanding the pool of the 20 IADL tasks

2. how to involve patients in selecting two relevant tasks of daily living from the pool of IADL tasks

3. how to invite the patient to perform the task

Interventionists of the **ELL **group and the substitutes will additionally learn

1. how each task is pre-structured in steps and how to give cues for these steps

2. how to anticipate possible errors of the patient before they occur and how to intervene and again give cues so that the patient is motivated to continue.

Interventionists of the **TEL **group and the substitutes will additionally learn

1. how to intervene when errors occur so that the patient is motivated to continue.

Seminars will be at different time points, in order to prevent contamination of specific seminar content between the ELL and TEL group. Two treatment manuals with detailed instructions will be provided, separately for the ELL and the TEL intervention.

Each interventionist of the ELL and the TEL condition and each substitute interventionist must apply 12 sessions to two pilot patients. The third and tenth relearning session with each pilot patient must be video recorded. The Dutch supervisors will use these video tapes to assess the quality of the treatment and to reflect on possibilities for improvement within the repetition seminar. Interventionists will receive certification based on the quality of their pilot treatment and their contribution within the repetition seminar. In order to monitor the treatment adherence during the study phase, the main ELL and TEL interventionist must provide video tapes with two tasks of the third and tenth relearning sessions with their first patient. The focus of the treatment adherence will be the interventionists' anticipation of and reaction on the patient's errors. Criteria will be specified in the treatment manuals. In cases of inappropriate treatment adherence the interventionist will receive an intensive telephone mentoring session. Continuing insufficient adherence will lead to the interventionist's replacement.

### Outcome

The primary outcome is the performance of the two selected and trained daily living tasks. The applied measurement is the Task Performance Scale. It demonstrated feasibility and responsiveness within the pilot trial. The evaluation of task performance by measuring the number of occurring errors or the number of insufficient task performances is an established standard in trials on relearning cognitive or daily living tasks. Patient's task performance will be video recorded by the interventionist, in order to assure blinded rating and to avoid that the patient will have potentially confusing contacts with several different persons. Blinded assessors from the REDALI-DEM headquarters in Freiburg will rate the videos regarding the number of occurring errors and steps performed insufficiently. Table 2 shows the primary and the secondary outcomes including measurement instruments and time points. Secondary outcome and control measures are performed by the study physician during the patient's visit at the study site, except the Verbal Rating Scale for the satisfaction with the intervention, which is applied by the interventionists at the patient's home.

**Table 2:** Measurement scheme including primary and secondary outcome

Weeks	0		11		16*		19-20		26
Measurement time points	t_0_		t_1_		t_2_				t_3_
Intervention		12 sessions		break		break	refresher	break	
Primary outcome									
Task Performance Scale (Rating of assessment video)	x		x		**x**				x

Secondary outcomes									
Satisfaction with intervention (Verbal Rating Scale)		each session					each session		
Activities of daily living (Interview for Deterioration in Daily Living Activities in Dementia)	x				**x**				x
Resource utilisation (Resource Utilisation in Dementia)	x				**x**				x

Control measurements									
Cognition (Mini Mental State Examination)	x				x				x
Course of Dementia (Clinical Dementia Rating)	x				x				x
Challenging behaviour (Neuropsychiatric Inventory, 12 items questionnaire)	x				x				x
Socio-demographic data	x								
Depression (Geriatric Depression Scale, 15 items)	x								
Attention (Trail Making Test)	x								
Intervention adherence (rating of treatment videos)		selected sessions							

* Primary measurement time point

### Sample Size

A sample size of 160 subjects (λ = 8.00, critical F = 3.901, numerator df = 1, denominator df = 158) is needed for the detection of small effects size (f = 0.10) in an analysis of variance with two groups and two repeated measurements at baseline and week 16 hypothesising an alpha of 5%, a power of 80% and a correlation of 0.6 between the measurement points.

#### Reasoning

**Figure 1:**
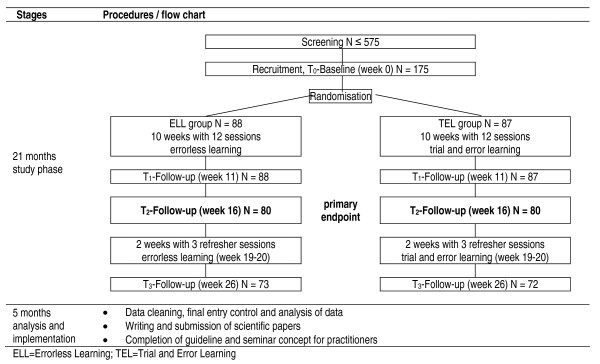
**Trial flow**. ELL = Errorless Learning; TEL = Trial and Error Learning.

The sample size of the Nijmegen pilot study was small (N = 12) [19]. Powerful within-subjects analyses revealed high effect sizes in post-treatment (η^2^_p _= 0.8, p < 0.01) and delayed assessment (η^2^_p _= 0.5, p = 0.01). In order to avoid bias from carry-over effects between the groups potentially occurring in a within-subjects design, in the present trial a between-subjects design will be conducted and the interaction effect between groups and measurement points T_0 _(Baseline) and T_2 _(16 weeks after baseline) will be analysed as primary endpoint. The between-subjects design will reduce the statistical power, consequently more subjects are needed. Furthermore, the correlation of r = 0.85 between the measurements in control and treatment groups as stated in the meta-analysis of Kessels et al. is quite large [18]. However, they excluded trials with between-subjects design and only analysed studies using within-subjects design. Considering that group differences might be influenced by several unknown confounders in our real-life setting at the patient's home we assume a correlation of r = 0.6 for the power calculation. Although the meta-analysis demonstrated large effect sizes (d = 0.87), it also found large confidence intervals (CI95% = 0.10-1.64). It is assumed that detecting even small effects is reasonable, because the implementation of relearning techniques will be a low-cost and a low-risk intervention. For these reasons, we performed a conservative power calculation, which is able to detect even small effect sizes for the interaction. The patients' planned trial flow is shown in Figure 1.

### Randomisation and Blinding

The trial statistician at a detached site will provide a 1:1 randomisation block-wise without stratification via e-mail for each individual case after recruitment and upon request of the site investigator. Independent assessors are blinded to group assignment. Following the nature of the intervention, participants are not blind for the type of intervention. However, neither the patients nor the interventionists will be explicitly presented an assumption as to which intervention may be more likely to improve activities of daily living. Moreover, both interventions include the same amount of personal involvement which prevents contrasting interventions with waiting group scenarios or grossly reduced personal involvement. Blinded assessment of the treatment effects will be achieved by videotaping the performance of the patient and removing all hints of prior treatment modality.

### Statistical analysis

Baseline measures and demographics of participants significantly differing at baseline in ELL and TEL group as well as control measures are used as co-variates in an analysis of variance. The primary intention-to-treat analysis will be compared with a per-protocol analysis.

The method "Last Observation Carried Forward" will not be used for the imputation of missing data, because it introduces bias in dementia research [20]. If necessary, the Full Information Maximum Likelihood method will be applied for data replacement [21,22]. Drop-out analysis will be performed comparing all baseline and control measurements and follow-up data of participants and dropouts as far as sufficient data are available. Therefore participants are asked to give consent to a final assessment using the Task Performance Scale at the dropout time point. Furthermore, the carers are asked to fill in the Interview for Deterioration in Daily Living Activities in Dementia at all measurement time points as planned.

The comparison of ELL and TEL group will be adjusted for baseline imbalance and process variations. Variations in treatment integrity, medication of achetylcholinesterase inhibitors, and study sites will be considered as possible mediating variables and analysed with hierarchical linear models. All statistical tests will be two-sided on an alpha level of 0.05.

### Ethical Considerations

In the trial and error condition, individuals will benefit from the trial and error learning, but will be exposed to the risk of experiencing deficits through insufficient task performances more frequently than individuals in the errorless condition. For cases where these experiences lead to severe behavioural disturbances the trial will be discontinued. If the ELL intervention proves to be superior to the trial and error learning, the errorless learning approach will reduce the experience of insufficient task performances in AD patients. In all cases, the closest caring relative of the patient will be asked to give assent to support the trial procedures by filling in forms and arranging appointments. Patients who are not able to consent will be included, if a legally appointed representative gives consent.

### Monitoring

Relevant Standardised Operating Procedures (SOP, http://www.tmf-ev.de/Produkte/SOP.aspx) will be adapted to the specific requirements of the REDALI-DEM study.

SOP-pre-visit: to ensure the reliable evaluation of recruitment capacity and competence of staff at each site

SOP-monitor-visit: to ensure the reliable data collection and transfer and appropriate query management at each study site

SOP-data-check: to ensure identical procedures, when incoming data are checked for correctness and queries are handled

SOP-data-entry-check: to ensure a rate of data entry errors of less than 0.2%

SOP-adverse-event: to ensure that adverse events are disclosed early and handled appropriately

### Safety

No evidence suggests that the application of learning strategies causes medical or mental instability in AD population. A trial steering committee will be constituted of two persons from the applicant group and one independent person with following responsibilities. (1) Statistician: supervision of statistics and assessment adherence monitoring. (2) Principal investigator from the Nijmegen research group: supervision of treatment adherence monitoring. (3) Professor in ethics: ethical judgement of the study protocol and possible adverse events. Severe adverse events are defined as non-elective hospital admissions or death. Adverse events will be reported according to the standards of Good Clinical Practice.

## Discussion

It is assumed that the technique of errorless learning may optimize procedural learning through intact implicit processing in AD patients [11-19,23]. Using this technique for relearning of ecological tasks is highly relevant for both, patients and carers, because it might enhance daily functioning and slow down the loss of the patient's autonomy. Deeper knowledge on effective teaching techniques and beneficial instructions in AD could have positive impact not only on health professionals but also on the interaction between patients, informal carers and voluntary helpers, which are increasingly deployed in dementia care.

About one third of patients with mild or moderate dementia receive informal care of 4 to 10 h per day and one third more than 10 h per day [24]. Our trial will also evaluate the impact of relearning selected ADL tasks on the informal care. We will try to collect separate data for times of actual help and of supervision, although we know from our former multi-centre RCT on psychosocial interventions in AD that carer have difficulties to differentiate these two kinds of informal care. If the stabilisation of the performance of two relevant daily living tasks could save about 1 h of informal care per day, the burden of care giving would be decreased by a minimum of 10%.

The trial results will inform us to improve the first existing evidence-based practice guideline for instructing individuals with neurogenic memory impairments [25]. An improved, user-friendly, and more detailed practice guideline will allow an efficient implementation of the implicit learning program for a wide range of care and service providers in dementia, such as physicians, occupational, physical and speech therapists, neuropsychologists, nurses, social workers, family carers and volunteers in health care.

## Abbreviations

AD: Alzheimer's Disease; ADL: Activities of Daily Living; ELL: Errorless Learning; MMSE: Mini Mental State Examination; TEL: Trial and Error Learning; REDALI-DEM: **Re**learning methods on **Da**ily **Li**ving task performance of persons with **Dem**entia

## Competing interests

The authors declare that they have no competing interests.

## Authors' contributions

SVR, RL, RK, MOR and MH contributed to study conception and design. SVR drafted the manuscript. RL, RK, MOR and MH revised the manuscript critically for important intellectual content. All authors read and approved the final manuscript.

## Pre-publication history

The pre-publication history for this paper can be accessed here:

http://www.biomedcentral.com/1471-2318/11/44/prepub
